# Overview of a Surface-Ripened Cheese Community Functioning by Meta-Omics Analyses

**DOI:** 10.1371/journal.pone.0124360

**Published:** 2015-04-13

**Authors:** Eric Dugat-Bony, Cécile Straub, Aurélie Teissandier, Djamila Onésime, Valentin Loux, Christophe Monnet, Françoise Irlinger, Sophie Landaud, Marie-Noëlle Leclercq-Perlat, Pascal Bento, Sébastien Fraud, Jean-François Gibrat, Julie Aubert, Frédéric Fer, Eric Guédon, Nicolas Pons, Sean Kennedy, Jean-Marie Beckerich, Dominique Swennen, Pascal Bonnarme

**Affiliations:** 1 INRA, UMR 782 Génie et Microbiologie des Procédés Alimentaires, F-78850, Thiverval-Grignon, France; 2 AgroParisTech, UMR 782 Génie et microbiologie des procédés alimentaires, F-78850, Thiverval-Grignon, France; 3 AgroParisTech, UMR 518 Mathématiques et Informatiques Appliquées, F-75231, Paris, France; 4 INRA, UMR 518 Mathématiques et Informatiques Appliquées, F-75231, Paris, France; 5 INRA, Institut Micalis, F-78352, Jouy-en-Josas, France; 6 AgroParisTech, Institut Micalis, F-78352, Jouy-en-Josas, France; 7 INRA, UR1404 Mathématiques et Informatique Appliquées du Génome à l’Environnement, F-78352, Jouy-en-Josas, France; 8 Actalia, F-74801, La Roche sur Foron, France; 9 INRA, US 1367 Metagenopolis, F-78352, Jouy-en-Josas, France; University of Naples Federico II, ITALY

## Abstract

Cheese ripening is a complex biochemical process driven by microbial communities composed of both eukaryotes and prokaryotes. Surface-ripened cheeses are widely consumed all over the world and are appreciated for their characteristic flavor. Microbial community composition has been studied for a long time on surface-ripened cheeses, but only limited knowledge has been acquired about its *in situ* metabolic activities. We applied metagenomic, metatranscriptomic and biochemical analyses to an experimental surface-ripened cheese composed of nine microbial species during four weeks of ripening. By combining all of the data, we were able to obtain an overview of the cheese maturation process and to better understand the metabolic activities of the different community members and their possible interactions. Furthermore, differential expression analysis was used to select a set of biomarker genes, providing a valuable tool that can be used to monitor the cheese-making process.

## Introduction

Microbial communities are of major importance in the fermentation of food products. Fermentation remains a widespread means for food processing and preservation, and fermented foods (including cheese) are widely consumed worldwide. The composition and behavior of the microbial communities in a cheese are important for its characteristic organoleptic properties, shelf life and safety [1]. These communities are involved in the generation of a wide range of diverse beneficial functions as a result of individual metabolism and/or complex ecological interactions [2].

To date, a plethora of work related to functions of technological interest have been published on cheese-inhabiting microorganism. Such studies mainly concern the ability of those microorganisms to generate functions such as proteolysis [3], lipolysis [4] and/or catabolic routes leading to aroma compound production [5–7]. However, although some microorganisms that inhabit cheese are known to be key drivers of the ripening process, our understanding of how individual microbes and microbial groups change over time within the cheese matrix and contribute to the structure and function of specific communities remains incomplete.

With the recent advances in high-throughput sequencing technologies (HTS), sensitive profiling of microbial communities from fermented food products can now be performed on an unprecedented scale via the massive sequencing of short DNA fragments [8,9]. Metagenomic studies, including both meta-barcoding (e.g., the deep-sequencing of variable regions of the prokaryotic SSU rRNA gene or of the fungal ITS) and whole metagenome sequencing projects, have made it possible to characterize the microbial community composition of many cheese varieties and to access the diversity of sub-dominant populations [10–13]. Furthermore, genome sequencing of several representative strains isolated from cheese or used as starter culture in the cheese-making process has allowed us to access their metabolic arsenal [14–16]. The next step towards a better understanding of how the cheese ecosystem functions would be to evaluate the expression of these genes *in situ*. As recently demonstrated for a Camembert-type cheese, this is feasible through metatranscriptomic analyses using RNA sequencing [17]. In this example, the authors followed the metabolic activity of a relatively simple community essentially composed of a yeast, *Geotrichum candidum*, and a fungus, *Penicillium camemberti*, and highlighted key functions and metabolic pathways that are expressed during the ripening process. Because the genome of these organisms were not available, *de novo* assembly of the RNA-Seq data (long reads) was required prior to functional assignment of the resulting contigs. This approach could be applied to more complex cheese microbial communities comprising both fungal and bacterial species. In this case, short reads analysis offering a higher sequencing depth is preferable, but it would be highly desirable to have the annotated reference genomes of all of the species.

In the present work, we combined microbiological, biochemical, metagenomic (DNA-Seq) and metatranscriptomic (RNA-Seq) data collected from a simplified microbial community capable of reproducing the complex metabolic pattern of cheese maturation [18,19]. To facilitate these analyses, we established a reference database of all the genomes of the studied community, onto which sequence reads could be mapped. The main objective of the study was to obtain a global view of the dynamics of the microbial community structure as well as the expression profiles of its metabolic potential throughout a ripening cycle at different scales—whole microbial community down to the gene level. Moreover, differential analysis of the ecosystem’s metatranscriptome was performed which should enable us to propose a set of biomarker genes that are representative of the most active species at various stages of ripening. Thus, we expect to reveal the sequential development and/or metabolic features of microbial species, and possibly highlight metabolic complementarities and possible interaction phenomena that sustain the expression of important functionalities of technological interest.

## Materials and Methods

### Cheese production

Full details on microorganisms used for cheese ripening and cheese production are given in the S1 File. Briefly, cheese production was performed with 120 L of pasteurized milk, under aseptic conditions in a sterilized chamber [20]. A lactic starter culture containing *Lactococcus lactis* subsp. *lactis* S3+ and S3- inoculated at concentrations equivalent to 2 x 10^6^ and 4 x 10^6^ CFU/mL, respectively, was used in combination with a mix of *Kluyveromyces lactis* 3550 (10^4^ CFU/mL), *Debaryomyces hansenii* 304 (10^4^ CFU/mL) and *Geotrichum candidum* ATCC 204307 (10^3^ CFU/mL). Next, 120 mL of a filter-sterilized CaCl_2_ solution (10%) and 40 mL of rennet (20 mg/L of chymosin (Chr. Hansen, Arpajon, France)) were added to allow the milk to coagulate. The curd was cut into small cheeses (diameter: 5 cm; height: 1.5 cm; weight: 26 g) and immersed in sterile brine (270 g/L NaCl, pH 5.5 measured using a contact electrode) to obtain a salt concentration of 1.7%. The five ripening bacteria (*Corynebacterium casei* UCMA 3821, *Brevibacterium aurantiacum* ATCC 9174, *Arthrobacter arilaitensis* CIP 108037, *Staphylococcus equorum* Mu2 and *Hafnia alvei* GB001) were inoculated onto the surface of the cheese at a rate of 2 x 10^5^ CFU/g. The inoculated cheeses were ripened for four weeks at 14°C and 97% relative humidity in sterile crystallizing basins.

### Cheese sampling and microbial analyses

Samples were collected on days 1, 7, 14, 21 and 31. Day 1 corresponds to the cheese curd (before immersion in brine). Three crystallizing basins (corresponding to three replicates) were analyzed at each time-point. As all cheeses were produced from the same batch of milk and inoculum, these replicates should not been considered as true biological replicates. The four small cheeses in each crystallizing basin were crushed and homogenized with sterile forks and knives. Serial dilutions were performed in 9 g/L NaCl from one gram of cheese and plated in triplicate on agar plates. Three selective culture media were used: brain heart infusion (Biokar Diagnostics) with amphotericin (50 mg/L) for cheese-surface bacteria, de Man-Rogosa-Sharpe (pH 6.5, Biokar Diagnostics) with amphotericin (50 mg/L) for lactic acid bacteria, and yeast extract-glucose-chloramphenicol (Biokar Diagnostics) with 2,3,5-triphenyltetrazolium chloride (10 mg/L) for yeasts. The strains could be selectively counted on these media because each had a distinct morphotype.

### Biochemical analyses

#### Lactose and lactate concentration

The lactose and lactate contents of cheeses were determined by high-performance liquid chromatography (HPLC), as previously described [20]. Briefly, a cheese suspension was prepared with finely ground cheese (7.5 g and 10 g for lactose and lactate, respectively) and 10 mL of distilled water. After incubation for 1 h at 50°C, it was homogenized for 2 min at 25000 rpm using a mechanical blender (Ultra-Turrax model T25, Ika Laortechnik).

For lactose measurement, 12.5 mL of Carrez I solution (150 g K_4_(Fe(CN)_6_), 3H_2_O /L water), 12.5 mL of Carrez II solution (240 g Zn(CH_3_COO)_2_, 2 H_2_O /L water) and 2.5 mL of 1 M NaOH were added to the suspension. After homogenization and incubation for 1 h at 25°C, the suspension was filtered through Whatman paper n°42 (GE Healthcare) and through a cellulose filter of 0.22 μm porosity (Minisart SP25, Sartorius). Finally, the filtrate was analyzed by HPLC.

For lactate measurement, 10 mL of 240 g/L trichloroacetic acid (TCA) and 10 mL of water were added to the suspension after cooling at 25°C. The mixture was incubated for 1h at 25°C and filtered through Whatman paper n°42 (GE Healthcare). Finally, the filtrate was analyzed by HPLC.

#### Free amino acids concentration

A cheese suspension was prepared from 5 g of cheese and 45 mL of distilled water. It was homogenized for 2 min at 25000 rpm using a mechanical blender (Ultra-Turrax model T25, Ika Laortechnik) and incubated for 30 min at 50°C. After centrifugation for 30 min at 5000 x g and 4°C, the supernatant was filtered through Whatman paper n°42 (GE Healthcare). The free amino acids concentration was measured on the filtrate according to the ninhydrin method [21] and quantified using leucine as a standard.

#### Proteolysis index

A cheese suspension was prepared from 10 g of cheese and 90 mL of distilled water. It was homogenized for 2 min at 25000 rpm using a mechanical blender (Ultra-Turrax model T25, Ika Laortechnik). This suspension was used for both total nitrogen and non-casein nitrogen content measure.

Total nitrogen content was measured directly on the cheese suspension using the Kjeldahl method according to standard NF EN ISO 8968–1.

The non-casein nitrogen content (soluble at pH 4.6) was measured according to the procedure described in standard NF ISO 27871. The cheese suspension was adjusted to pH 4.6 using 5N HCl and filtered through Whatman paper n°42 (GE Healthcare). The nitrogen content of this soluble fraction was measured using the Kjeldahl method according to standard NF EN ISO 8968–1.

The proteolysis index was calculated by dividing the soluble nitrogen content by the total nitrogen content and multiplying by 100, as previously described [22].

#### Lipolysis index

The lipolysis index corresponds to the quantity of KOH (in mg) required to neutralize the free fatty acids contained in 1 g of cheese. It was determined by adapting the titrimetric measurement described by Mouillet et al. [23]. Briefly, 2.5 g of cheese was homogenized for 2 min at 25000 rpm using a mechanical blender (Ultra-Turrax model T25, Ika Laortechnik) in 22.5 mL of ethanol/diethyl ether (1:1 v/v) in order to extract fat content. The suspension was filtered through Watman paper No. 42 (GE Healthcare) and the acidity of the filtrate was measured by standard titration with 1M KOH in ethanol using phenolphthalein as a color indicator.

### Metagenomic and metatranscriptomic analyses

#### Extraction of DNA from cheese samples

Total genomic DNA was obtained after casein solubilization and cell recovery from two grams of cheese by adapting the method described by Baruzzi et al. [24]. Cheese samples were mixed with 18 mL of sodium citrate solution (20 g/L trisodium citrate dihydrate) and the mixture was dispersed for 2 min at 24,000 rpm with a mechanical blender (Ultra-Turrax model T25, Ika Laortechnik). A second dispersion was performed after 10 min of incubation at room temperature. The mixture was then centrifuged at 6,400 x g for 10 min at 4°C, and the supernatant was removed. The casein pellet (containing the microbial cells) was resuspended in 5 mL of a Triton X-100 aqueous solution (2.5% v/v), vigorously shaken, heated in a water bath at 70°C for 10 min, centrifuged at 6,400 x g for 10 min at 4°C, and rinsed twice in physiological saline solution. The pellet was dissolved in a mixture of 270 μL of guanidium thiocyanate (4 M) in Tris-HCl (pH 7.5, 0.1 M) and 30 μL of sodium lauroyl sarcosinate (100 g/L), and transferred to a 2 mL tube containing 250 mg of 0.1 mm-diameter zirconium beads and 250 mg of 0.5 mm-diameter zirconium beads (Sigma, St-Quentin-Fallavier, France). Proteinase K treatment, bead-beating, phenol-chloroform extraction, RNase treatment and ethanol precipitation were then performed as previously described by Leclercq-Perlat et al. [25]. The DNA pellet was dissolved in 120 μL of Tris EDTA buffer (10 mM Tris-HCl, pH 8.0, 1 mM EDTA).

#### Extraction of RNA from cheese samples

Total RNA was extracted from 500 mg cheese samples without the prior separation of microbial cells, as previously described [26]. Three separate extractions were performed for each cheese sample and pooled before library construction. The quality of total RNA was analyzed with a 2100 Bioanalyzer and RNA 6000 NANO chips (Agilent, Palo Alto, CA, USA).

#### DNA and cDNA library construction and SOLiD sequencing

Library construction and SOLiD sequencing were performed at the INRA MetaQuant facility (Jouy-en-Josas, France). The DNA libraries were constructed from 3 μg of total DNA by using the SOLiD Fragment Library Construction Kit (Applied Biosystems, Bedford, MA, USA) and then barcoded with the SOLiD Fragment Library Barcoding Kit. DNA samples collected at day 1 did not allow to construct libraries that meet the quantity and quality criteria enabling to perform sequencing and, thus, were excluded from this study. The cDNA libraries were constructed from 200 to 500 ng total RNA using a SOLiD Whole Transcriptome Analysis Kit and were barcoded with the SOLiD Transcriptome Multiplexing Kit. The SOLiD ePCR kit and SOLiD Bead Enrichment Kit were used to process DNA and cDNA samples for sequencing, and the SOLiD 4 System was used for sequencing.

#### Reads processing and mapping

Short sequence reads of 35 bp were mapped (a maximum of three mismatches were allowed) onto a reference composed of the nine sequenced strains of microorganisms (Table 1) using Bowtie software (version 0.12.7) [27].

**Table 1:** Reference genomes used for mapping of the sequence reads.

Species	Nb of genes (CDS)	Genome size (Mb)	Status	BioProject Accession number	Reference
**Six bacteria**					
*Arthrobacter arilaitensis* CIP 108037	3,689	3.92	Complete	PRJEA50353	[40]
*Brevibacterium aurantiacum* ATCC9174	4,105	4.39	Draft (76 contigs)	PRJNA405	no reference
*Corynebacterium casei* UCMA3821	3,015	3.12	Draft (106 contigs)	PRJEA76363	[41]
*Hafnia alvei* GB001	4,692	4.89	Draft (137 contigs)	PRJEB6257	to be published
*Lactococcus lactis* S3	2,615	2.49	Draft (163 contigs)	PRJEB6259	to be published
*Staphylococcus equorum* Mu2	2,932	2.96	Draft (30 contigs)	PRJEA88899	[76]
**Three yeasts**					
*Debaryomyces hansenii* CBS767	6,421	12.2	Complete	PRJNA13832	[14]
*Geotrichum candidum* ATCC204307	6,958	24.9	Complete	PRJEB5752	to be published
*Kluyveromyces lactis* NRRL Y1140	5,120	10.8	Complete	PRJNA13835	[14]

After the smart filtering of multiple reads, the numbers of reads mapped onto the reference were counted with METEOR software [28]. Only uniquely mapped reads were further analyzed. General library statistics are available in S1 Table.

#### DNA-Seq data analyses

For each microorganism, sequencing coverage was estimated with the Lander/Waterman equation [29]: C = LN / G, where C is the coverage, L is the read length, N is the number of reads and G is the haploid genome length.

#### RNA-Seq data analyses

Sequence reads mapping to the coding DNA sequence (CDS) features were retrieved from the raw dataset.

First, functional classification of the metatranscriptomic dataset was performed using the Kyoto Encyclopedia of Genes and Genomes (KEGG) annotations [30]. To do this, data were filtered to remove genes displaying an average of less than five reads per sample across the entire dataset (15 samples) and normalized according to the library size using custom scripts built under the statistical environment R (http://www.r-project.org/).

Second, differential expression analysis over time was done by comparing the number of mapped reads for each gene at two different time-points (n = 3 per ripening time). For this analysis, data (six samples for each comparison) were filtered: (i) genes with less than five mapped reads were eliminated; and (ii) genes whose replicates were heterogeneous were discarded (variation coefficient > minimum mean observed for the two groups of three replicates). Data normalization and determination of differentially expressed genes were then conducted using the Bioconductor DESeq2 package in the statistical environment R [31,32]. Raw p-values were adjusted for multiple testing using the Benjamini-Hochberg procedure [33], which assesses the False Discovery Rate. Gene transcripts with an adjusted p-value < 0.05 were considered to be differentially abundant between two ripening times.

### Sequence accession numbers

The raw SOLiD read data for all samples was deposited in the European Bioinformatics Institute's European Nucleotide Archive under the accession number PRJEB6315. Accession numbers of reference genomes are given in Table 1.

## Results and Discussion

### Overview of the cheese microbial community composition and activity during ripening

**Fig 1:**
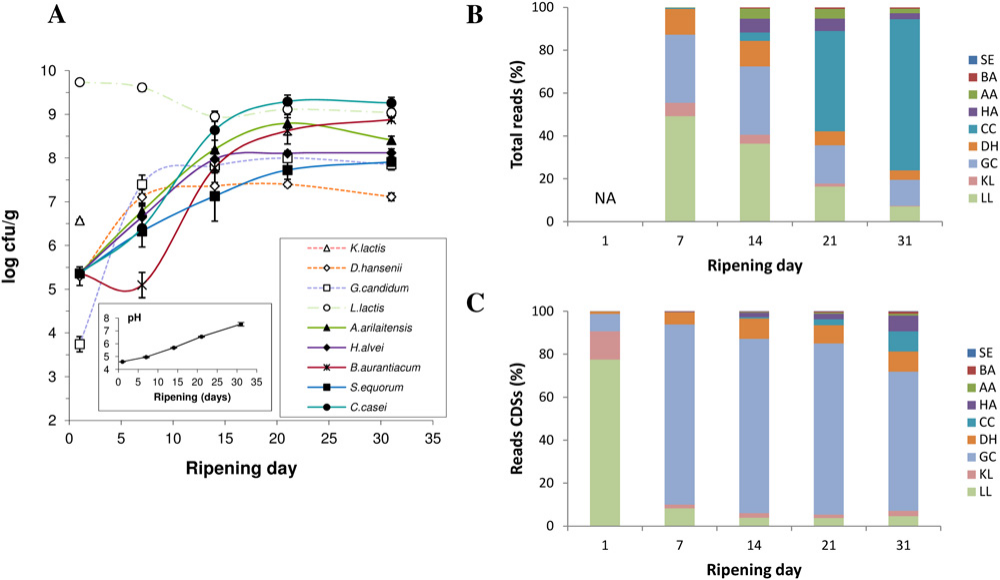
Changes in the microbial community structure during surface-ripened cheese maturation. (A) Microbiological counts and pH measurements. (B) Distribution of metagenomic data by species. (C) Distribution of metatranscriptomic data by species (only reads mapping CDS features were taken into account). SE: *Staphylococcus equorum*. BA: *Brevibacterium aurantiacum*. AA: *Arthrobacter arilaitensis*. HA: *Hafnia alvei*. CC: *Corynebacterium casei*. DH: *Debaryomyces hansenii*. GC: *Geotrichum candidum*. KL: *Kluyveromyces lactis*. LL: *Lactococcus lactis*. NA: data not available.

In order to estimate the overall contribution of each microorganism throughout the cheese-ripening kinetics, we used three complementary approaches: microbiological counting providing a measurement of the viable cells, metagenomic sequencing (DNA-Seq) indicating the proportion of DNA molecules from both viable and dead cells in each sample, and metatranscriptomics (RNA-Seq) reflecting the active populations (Fig 1).

Growth measurements revealed that the early development of yeasts, especially *D*. *hansenii* and *G*. *candidum*, contributed to the increase in the pH of the cheese curd (Fig 1A), which, in turn, prompted the development of acid-sensitive bacteria (*A*. *arilaitensis*, *H*. *alvei*, *B*. *aurantiacum*, *S*. *equorum*, *C*. *casei*). The lactic acid bacterium *L*. *lactis* was the most prominent bacterial population until day 14, but did not grow during the ripening process. *Kluyveromyces lactis* was detected at day 1 but was not detectable thereafter. *C*. *casei* was the dominant bacterium at the end of the ripening process (~10^9^ CFU/g of cheese). *Geotrichum candidum* was the major yeast at day 7. At the end of the ripening process (day 31), the populations of *G*. *candidum* and *D*. *hansenii* were 7 x 10^7^ CFU and 1 x 10^7^ CFU/g of cheese, respectively. The pH of the cheese curd increased from 4.6 at the beginning of ripening to 7.5 at day 31.

Similar trends were observed between microbiological counts and DNA-Seq data (Fig 1B). The proportions of reads attributed to each microorganism revealed a co-dominance of *L*. *lactis* and yeasts during the first two weeks of the ripening process, which were progressively replaced by surface bacteria, principally *C*. *casei* (71% of the total reads at day 31). *S*. *equorum* exhibited the lowest genome coverage in our dataset with a maximum of 0.12 X at day 14 (Table 2). In contrast, abundant species such as *L*. *lactis* and *C*. *casei* showed the highest genome coverages with 218 X for the former (day 7) and 176 X for the latter (day 31). The most abundant yeast, *G*. *candidum*, displayed a maximum coverage of 14 X at day 7. The differences observed between CFU counting (Fig 1A) and the proportions of DNA-Seq reads per species (Fig 1B) might be due to several reasons. First, CFU counting is supposed to measure living cells whereas DNA sequencing target both living and dead cells. Second, CFU counting may provide an altered view of the living community structure because of viable but nonculturable (VBNC) issues, the number of individual cells generating every colonies or the difficulty to count some species (such as *G*. *candidum* which is a filamentous yeast). Third, DNA extraction bias (not assessed in this study), especially the cell separation step, as well as genome size may influence the proportion of reads by species observed with DNA-Seq.

**Table 2:** Sequencing coverage (C) and percentage of genes (P) with at least an average of five uniquely mapped reads in the DNA-Seq dataset across the three replicates for each microbial genome during ripening.

Species^a^	Day 7		Day 14		Day 21		Day 31	
	C	P	C	P	C	P	C	P
AA	0.52	81.4%	11.53	98%	8.39	97.8%	4.13	97%
BA	0.03	0.3%	1.02	93.1%	1.05	93.1%	0.99	92.8%
CC	1.35	93.6%	11.54	96.3%	110.59	96.8%	175.54	96.8%
DH	10.90	98.6%	9.04	98.5%	3.63	98.3%	2.78	98%
GC	14.39	100%	12.16	100%	4.99	100%	3.78	100%
HA	0.21	42.1%	12.25	99.9%	8.47	99.8%	4.40	99.5%
KL	6.29	97.1%	3.54	97.1%	0.86	96.3%	0.26	75.5%
LL	218.17	99.5%	134.74	99.5%	43.40	99.5%	21.31	99.3%
SE	0.04	0.2%	0.12	13.7%	0.11	12.4%	0.09	7.8%

^a^AA = *Arthrobacter arilaitensis*; BA = *Brevibacterium aurantiacum*; CC = *Corynebacterium casei*; DH = *Debaryomyces hansenii*; GC = *Geotrichum candidum*; HA = *Hafnia alvei*; KL = *Kluyveromyces lactis*; LL = *Lactococcus lactis*; SE = *Staphylococcus equorum*

RNA-Seq data indicated that *L*. *lactis* and *K*. *lactis* were the most active species at day 1 (77% and 13% of total CDS reads, respectively) (Fig 1C). The proportion of reads from *G*. *candidum* accounted for 84% of total CDS reads at day 7 and remained highly dominant over time (with 65% of total CDS reads at day 31). Read counts corresponding to *D*. *hansenii* transcripts increased from day 1 (1%) to day 14 (9%) and remained stable thereafter. Ripening bacteria, especially *C*. *casei* and *H*. *alvei*, were mainly detected at the end of ripening with RNA-Seq (9% and 7% of the CDS reads at day 31, respectively). Again, RNA extraction efficiency might be variable between microorganisms (especially between eukaryotes and prokaryotes) and maybe also between ripening times. This may have influenced the distribution of reads observed with this dataset.

Overall, the results highlighted the successive development and metabolic activity of different microbial groups, *L*. *lactis* and *K*. *lactis* at the beginning of cheese maturation, followed by *G*. *candidum* and *D*. *hansenii* and, finally, acid-sensitive bacteria. This dynamics is in accordance with other studies conducted on surface-ripened cheese, which depicted a similar development sequence of the microbial species [19,34,35]. However, we provide new information here with respect to the metabolic activity of the different cheese-ripening populations.

### Functional expression of the cheese ecosystem over time

**Fig 2:**
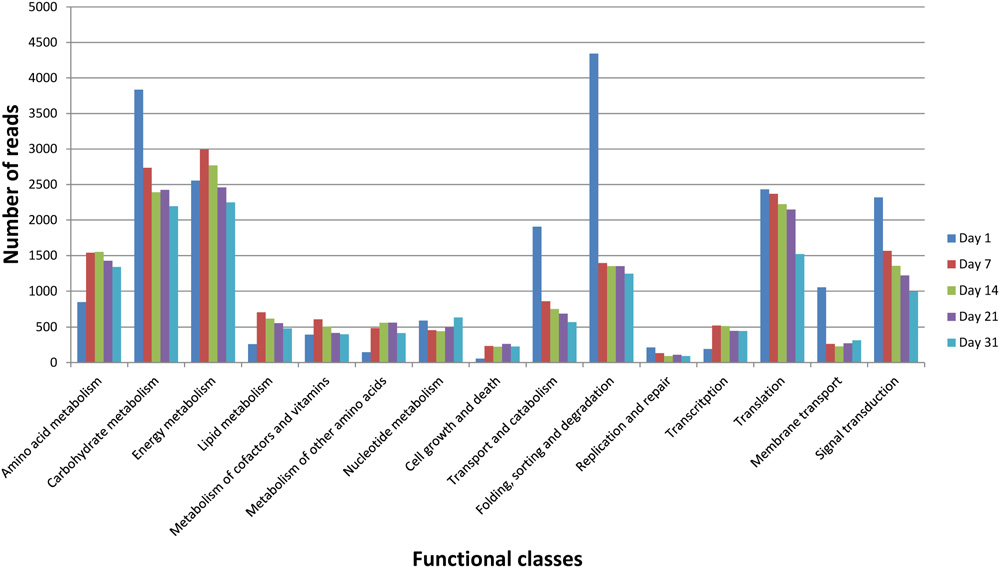
Functional classification of the metatranscriptome during surface-ripened cheese maturation. Functional classes were determined according to KEGG annotations. Read counts corresponding to all species were cumulated. Read numbers were normalized (according to the library size) to 50,000 reads per sampling day.

The global expression pattern of the cheese ecosystem throughout the ripening process was evaluated by normalizing all data by library size (S2 Table) and classifying RNA-Seq reads according to KEGG annotations (Fig 2). Data were also mapped onto the KEGG general map to obtain a dynamic view at the ecosystem level and per microbial species (S1 Fig). The KEGG categories—Amino acid metabolism, Carbohydrate metabolism, Energy metabolism, Transport and catabolism, Folding, sorting and degradation, and Translation and Signal transduction—accounted for the most abundant transcripts throughout the ripening period. These data are consistent with those observed on a Camembert-type cheese using a similar approach [17]. Differences in the expression dynamics between the functional classes were observed. However, this possibly reflected changes of several distinct metabolic pathways and required a more detailed analysis. Furthermore, it should be noticed that a great proportion of reads (between 69 and 81% depending on the sample) mapped genes without ortholog in the KEGG database. This included both annotated genes such as those encoding non enzymatic proteins or enzymes not yet referenced in KEGG pathways, as well as unknown genes.

#### Energy metabolism and iron homeostasis

The great majority of genes involved in energy production detected in our metatranscriptomic dataset were involved in the oxidative phosphorylation pathway (PATH:ko00190) (S2 Fig). As expected, the dynamics in the detection of transcripts involved in this pathway followed the changes in the community structure observed using microbial counts (Fig 1A). *G*. *candidum* genes encoding ATPases (EC:3.6.3.14 and 3.6.3.6), NADH dehydrogenase (EC:1.6.5.3) and cytochrome C oxidase (EC:1.9.3.1) accounted for the most frequently detected genes (S2 Table). Their maximum detection levels were observed between days 7 and 21. At day 1, it was possible to observe transcripts of genes encoding *K*. *lactis* ATPase (EC:3.6.3.6) and *L*. *lactis* ATPase (EC:3.6.3.14), NADH dehydrogenase (EC:1.6.99.3) and cytochrome D ubiquinol oxidase (EC:1.10.3.-), which reflected the early growth of these two microbial species. At the late stage of ripening (days 21 to 31), the detection of gene transcripts involved in the oxidative phosphorylation pathway from *H*. *alvei*, *C*. *casei* and, to a lesser extent, *A*. *arilaitensis* increased.

Metals ions, especially iron, are cofactors and/or components of various enzymatic systems involved in key microbial metabolisms (e.g., respiration). In cheese, iron often forms complexes with various curd components such as proteins (e.g., lactoferrin, ferritin and casein) and peptides, leading to low bioavailability [36]. Eukaryotic and prokaryotic microorganisms have different specific systems to transport metals. Numerous bacteria synthesize and excrete siderophores to trap iron [37]. However, some yeasts, including *K*. *lactis*, *D*. *hansenii* and *G*. *candidum*, express different siderophore transport systems to take advantage of siderophores present in their environment [38,39]. Another interesting feature is that fungal genes encoding iron high-affinity transport systems are generally induced under iron starvation conditions [38]. In our metatranscriptome, gene transcripts related to iron capture and/or transport were frequently detected (S2 Table), which may indicate a need for iron mobilization within the microbial community. For instance, *feoB* transcripts encoding a ferrous iron transport protein in *L*. *lactis* were mainly detected at day 1, transcripts of SIT1 encoding a ferrioxamine B transporter in *G*. *candidum* were essentially detected from day 7 to day 21, and transcripts of several iron-siderophore ABC transporters from *C*. *casei* (locus tag CCAS_01175, CCAS_03565, CCAS_00130, CCAS_05220, CCAS_05225, CCAS_12545, CCAS_12550, CCAS_12555, CCAS_10415) were mainly detected at day 31. Furthermore, sequence reads mapping to genes encoding high-affinity iron transport systems from both *G*. *candidum* (e.g., FTR1, FET3, FTH1), *D*. *hansenii* (e.g., FET3) and *K*. *lactis* (e.g., FTH1) were also detected in our conditions. Surprisingly, we did not observe bacterial transcripts for genes possibly involved in siderophore biosynthetic pathways, although *A*. *arilaitensis*, *C*. *casei* and *B*. *aurantiacum* are known to carry such pathways in their genome [26,40,41]. This could be due to an insufficient sequencing depth for cheese-surface bacterial species. A second hypothesis, which requires complementary analysis to be confirmed, could be that peptides released during the ripening process through casein hydrolysis would act as iron trappers, as suggested by [36], and be used as siderophore-like complexes by members of the microbial community.

#### Lactose consumption

**Fig 3:**
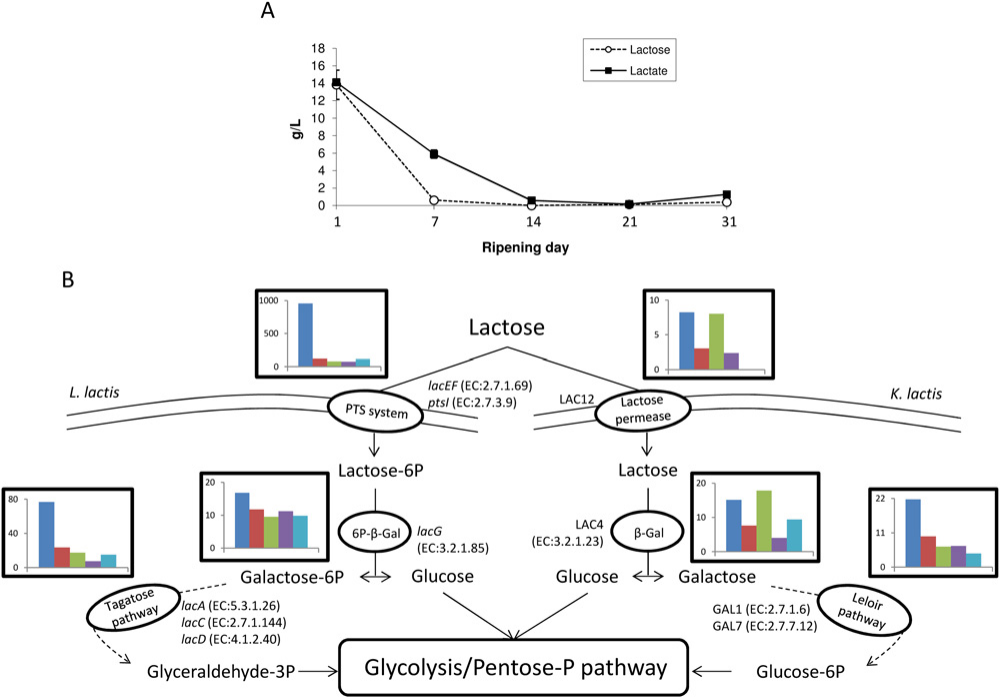
Lactose metabolism during surface-ripened cheese maturation. (A) Lactose and lactate concentrations. (B) Expression dynamics of lactose degradation pathways in *Lactococcus lactis* and *Kluyveromyces lactis*. Read numbers were normalized (according to the library size) to 50,000 reads per sampling day. For each degradation step, a histogram represents cumulative read numbers when several genes were involved.

According to Fig 2, carbohydrate metabolism was mainly detected in the early stage of ripening. Lactose is the major carbohydrate compound present in cheese curd and is rapidly consumed during the process (Fig 3A). We identified numerous expressed genes encoding enzymes involved in lactose uptake and degradation (Fig 3B and S3A Fig). For example, *L*. *lactis* genes encoding components of the lactose-specific phosphoenolpyruvate-dependent transport system (PTS), including *lacEF* (EC:2.7.1.69) and *ptsI* (EC:2.7.3.9), were highly expressed at day 1. The PTS system allows the concomitant translocation and phosphorylation of carbohydrates in *L*. *lactis*. The detection of components from other carbohydrate-specific PTS systems such as *celB* (EC:2.7.1.69) and *manXYZ* (EC:2.7.1.69) might indicate a versatile use of these systems for lactose uptake in this bacterium, as previously suggested by Aleksandrzak-Piekerczyk [42]. Transcripts of the *lacG* gene encoding the 6P-β-Galactosidase (EC:3.2.1.85), as well as *lacACD* (EC:5.3.1.26; EC:2.7.1.144; EC:4.1.2.40) involved in Lactose-6-P degradation via the D-Tagatose-6-P pathway in *L*. *lactis*, were also detected, mainly during the early stages of ripening (days 1 to 7). The products from this pathway, i.e., β-D-Glucose and Glyceraldehyde-3-P, then undergo glycolysis and/or the pentose phosphate pathway. Transcripts involved in these pathways were also highly detected in *L*. *lactis* at the same time (S2B and S2C Fig).

*K*. *lactis* is also able to metabolize lactose, but this yeast uses a slightly different pathway [43]. Lactose is imported through a specific lactose permease (encoded by the gene LAC12) and is then metabolized by a β-Galactosidase (EC:3.2.1.23) encoded by LAC4 and enzymes involved in the Leloir pathway (EC:2.7.1.6; EC:2.7.7.12) and encoded by genes GAL1 and GAL7. The expression of all genes from *K*. *lactis* involved in this pathway was also observed in our dataset, their detection being the most important during the early stage of ripening (days 1 to 14) (Fig 3B).

*G*. *candidum* does not consume lactose [44], whereas *D*. *hansenii* can efficiently catabolize this disaccharide [22]. Although our analysis indicated the detection of transcripts corresponding to the KEGG galactose pathway (PATH:ko00052) in these species (S3A Fig), this mainly corresponded to the detection of genes encoding enzymes not necessarily specific to this pathway, such as phosphoglucomutase (EC:5.4.2.2), 6-phosphofructokinase (EC:2.7.1.11) and hexokinase (EC:2.7.1.1). Indeed, these enzymes might instead reflect the detection of glycolysis (PATH:ko00010, S3B Fig) and the pentose phosphate pathway (PATH:ko00030, S3C Fig). Consequently, these species are not likely to significantly contribute to the lactose degradation in our model cheese.

Together, these results highlighted that in our experimental conditions, one of the key functions sustaining the cheese-ripening process, namely lactose biodegradation, involved the active participation of both *L*. *lactis* and *K*. *lactis* and revealed a functional redundancy existing for this metabolism within the studied ecosystem, as suggested from previous work [45].

#### Lactate metabolism

Lactate is of major importance in cheese making. It is produced from lactose present in milk by lactic acid bacteria (Fig 3A). Two lactate dehydrogenases transcripts of *L*. *lactis*, *ldhA* (EC:1.1.1.27) and *dld* (EC:1.1.2.4), were generally detected at day 1 (S2 Table), suggesting that *L*. *lactis* actively produces lactate at the early stage of ripening. Lactate exporter(s), responsible for lactate extrusion from the intracellular environment in *L*. *lactis*, have not yet been characterized. Carvalo et al. [46] proposed llmg_2513 CDS from *L*. *lactis* MG1363 as a good candidate to encode a lactate transporter based on its predicted protein sequence topology that is similar to known lactate transporters from other species (such as LldP from *E*. *coli*, YqkI from *B*. *subtilis* and JEN1 from *S*. *cerevisiae*), but failed to confirm this activity using the mutagenesis approach. LLACS3_00055 from *L*. *lactis* S3, used as starter culture in our experiment, the only CDS presenting a strong homology with llmg_2513 (89% sequence identity at the nucleic level), was not detected in our metatranscriptome and, thus, would probably not be responsible for lactate export. Several gene transcripts encoding transporters in *L*. *lactis* were although detected, mostly at day 1, including MFS and ABC family transporters that could be good candidates to facilitate the movement of small solutes such as lactate across cell membranes. However, none of them could be identified as a potential lactate transporter on the basis of sequence similarity with known sequences from other organisms.

Lactate degradation by the cheese microflora, principally yeasts, is a key driver for curd deacidification and occurred essentially during the two first weeks of ripening in our conditions (Fig 3A). Concomitantly, we detected reads for genes JEN1, encoding a lactate transporter, together with DLD2 (EC:1.1.2.4), encoding a lactate dehydrogenase, in both *D*. *hansenii* and *G*. *candidum* (S2 Table). It should also be mentioned that *G*. *candidum* DLD1 transcripts encoding a D-lactate dehydrogenase (EC:1.1.2.4) and CYB2 encoding a L-lactate dehydrogenase (EC:1.2.2.3) were also detected, generally between day 7 and day 21. In *Saccharomyces cerevisiae* [47,48] and *D*. *hansenii—*cultivated in a cheese-like medium [49]—the authors reported that DLD1 and CYB2 were induced by lactate, making them good candidates for lactate degradation.

#### Protein and amino acid degradation

**Fig 4:**
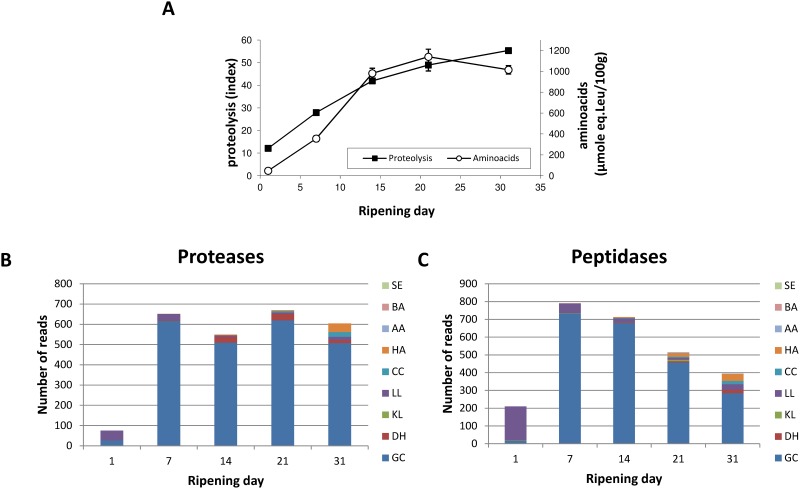
Protein degradation during surface-ripened cheese maturation. (A) Proteolysis and free amino acid concentration. Expression data observed for genes encoding proteases (B) and peptidases (C). Read numbers were normalized (according to the library size) to 50,000 reads per sampling day. SE: *Staphylococcus equorum*. BA: *Brevibacterium aurantiacum*. AA: *Arthrobacter arilaitensis*. HA: *Hafnia alvei*. CC: *Corynebacterium casei*. LL: *Lactococcus lactis*. KL: *Kluyveromyces lactis*. DH: *Debaryomyces hansenii*. GC: *Geotrichum candidum*.

Proteins and, more precisely, caseins, are major carbon and energy sources for microbial species living on cheese, together with lactose, lactate and lipids [6]. Proteolysis, which refers to the cleavage of caseins into small peptides and, ultimately, free amino acids by microbial proteases and peptidases, took place progressively and steadily in cheese, as attested to by the evolution of the proteolysis index and free amino acid concentrations (Fig 4A). Because a specific category for proteolysis does not exist in the KEGG database, we manually built two enzyme classes (Proteases and Peptidases) based on gene product annotations present in the reference genomes, and analyzed their expression profile during cheese ripening (Fig 4B and 4C). Normalized expression data for all these genes along with their annotation is given in S3 Table. These results suggested that *G*. *candidum* was the major contributor to proteolysis in this simplified surface-ripened cheese and supported other studies that indicated that proteolysis mainly occurs during the first three weeks of soft cheese ripening [17,50].

In the genome of *G*. *candidum*, only one predicted CDS encoding a putative protease contains a signal sequence indicating a possible extracellular localization according to SignalP [51] and the PSORT server [52]. However, we didn’t detected any read mapping to this CDS in our metatranscriptome. Similar genome analysis revealed that there is no extracellular protease encoding genes predicted in the *D*. *hansenii* and *K*. *lactis* available genomes, but contrary to *Y*. *lipolytica*, another yeast commonly found at the surface of cheese. Furthermore, extracellular protease activity assay performed using the strains used for our experimental cheese production supported those predictions (S4 Fig). Thus, we hypothesized that there were three possible non-exclusive ways for *G*. *candidum* to utilize caseins. First, it may directly uptake casein-derived peptides present in the extracellular environment, freed from the hydrolytic activities of both rennet used for milk coagulation [53] and *L*. *lactis* used as starter culture [54]. In our metatranscriptomic dataset, this was supported by the high number of reads observed for OPT2 and GAP1 genes of *G*. *candidum* (S2 Table), mainly between days 7 and 21, encoding an oligopeptide transporter and a general amino acid permease, respectively, which are known to contribute to the amino acid and protein uptake in yeasts [55,56]. Furthermore, several transcripts reflecting the proteolytic activity of *L*. *lactis* were detected earlier in the ripening kinetics, between days 1 to 7, namely *pepA*, *pepDB*, *pepC*, *pepN*, *pepM*, *pepT*, *pepV* and *pepX* encoding various peptidases (EC:3.4.11.7; EC:3.4.11.23; EC:3.4.22.40; EC:3.4.13.-; EC:3.4.11.2; EC:3.4.11.18; EC:3.4.11.4; EC:3.4.14.11) involved in the intracellular cleavage of small peptides into free amino acids [54] as well as genes *optA*, *optC* and *optF* encoding an oligopeptide ABC transporter system that has been shown to be responsible for casein-derived peptide transport for amino acid supply in *L*. *lactis* [57]. Second, *G*. *candidum* may internalize caseins to the vacuole by endocytosis and degrade them using vacuolar proteases and peptidases. In agreement with this hypothesis, expression data revealed the important detection as of day 7, of several genes involved in endocytosis in *G*. *candidum*, among which the most frequently detected ones were EDE1 encoding an endocytic protein [58], SAC6 encoding fimbrin [59], ALY2 encoding an alpha arrestin [60], and PIL1 and LSP1 encoding primary components of eisosomes [61]. Among the most frequently transcribed genes encoding proteases and peptidases, CPS1 (EC:3.4.17.4), PRC1 (EC:3.4.16.5) and PEP4 (EC:3.4.23.25) encode enzymes known to be active in the vacuole [62]. The third possible way that casein can be used by *G*. *candidum* is the liberation of cellular proteases and peptidases in the extracellular environment during cell lysis, e.g., the highly expressed metalloendopeptidase encoded by the PRD1 gene. Although no data in our metatranscriptomic dataset enable us to support this hypothesis, this mechanism has already been suggested for a cheese isolate of *D*. *hansenii* [63].

**Fig 5:**
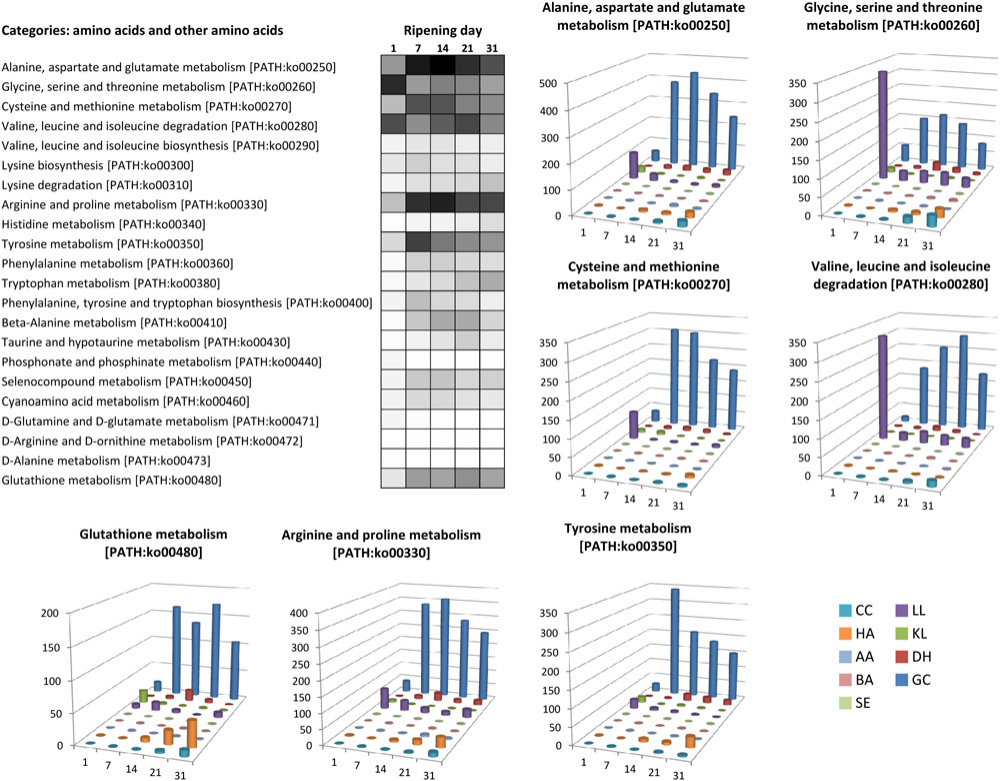
Gene expression related to amino acid metabolism. For each pathway, the heatmap represents the expression dynamics over time (cumulative number of normalized reads per pathway) using a gray scale bar from 0 read in white to 500 reads in black. For seven pathways, histogram charts detail this dynamic per microbial species. CC: *Corynebacterium casei*, HA: *Hafnia alvei*, AA: *Arthrobacter arilaitensis*, BA: *Brevibacterium aurantiacum*, SE: *Staphylococcus equorum*, LL: *Lactococcus lactis*, KL: *Kluyveromyces lactis*, DH: *Debaryomyces hansenii*, GC: *Geotrichum candidum*.

The next step following initial proteolysis is amino acid biodegradation, which is generally linked to the cheese matrix alkalinization and volatile compound production known to play an important role in aroma perception [5,6,64–66]. The most dominant amino acids composing caseins are glutamate, proline, leucine, lysine, aspartate, valine, serine, tyrosine and isoleucine. As shown in Fig 5, among the complete list of amino acid metabolic pathways, those responsible for the metabolism of these dominant amino acids are also the most detected in our metatranscriptome. *G*. *candidum* accounted for the majority of the expression data observed regarding most amino acid metabolism. For example, genes involved in glutamate catabolism were highly detected in this organism. This included genes encoding the NAD-dependent glutamate dehydrogenase (GDH2, EC:1.4.1.2) and the NADP-dependent glutamate dehydrogenase (GDH3, EC:1.4.1.4) responsible for the deamination of this amino acid to generate 2-oxoglutarate, which is then supplied to the TCA cycle, as well as genes encoding the glutamate decarboxylase (GAD1, EC:4.1.1.15), degrading glutamate into 4-aminobutyrate (GABA), which is then converted into succinate by the enzymes 4-aminobutyrate aminotransferase (EC:2.6.1.19) and NAD(P)-dependent succinate semialdehyde dehydrogenase (EC:1.2.1.79), encoded by genes UGA1 and UGA2, respectively, which also feed the TCA cycle. GDH2 induction has already been observed in *G*. *candidum* by RT-qPCR in Reblochon-type cheese (a French surface-ripened cheese) at the end of ripening, and this gene was proposed as a biomarker for amino acid catabolism [67]. However, *G*. *candidum* was not the only microorganism responsible for amino acid catabolism in our experimental surface-ripened cheeses. Transcripts involved in glycine, serine and threonine metabolism (PATH:ko00260) as well as the valine, leucine and isoleuline degradation pathway (PATH:ko00280) were also highly detected in *L*. *lactis* at day 1.

#### Lipid metabolism

Lipolysis refers to the hydrolysis of triglycerides and results in the liberation of free fatty acids (FFAs) that are important precursors of catabolic reactions that produce volatile compounds that contribute to cheese quality and flavor [4,66,68]. In our metatranscriptomic data, we observed that lipid metabolism pathway expression approximately followed the same dynamics as amino acid metabolism, i.e., a strong increase from day 1 to day 7 followed by a global stability or a slow decrease during the next three weeks (Fig 2). This is in agreement with the dynamics observed by Lessard et al. [17] on a Camembert-type cheese. Furthermore, in our study, the lipolysis index steadily increased along the ripening process, which indicates a regular lipolysis of the food matrix (S5 Fig). *G*. *candidum* once again accounted for the most highly expressed genes detected for this metabolism and is likely to be the major contributor to cheese lipolysis in our conditions. However, we also detected two genes encoding putative esterases from *L*. *lactis* (locus tag: LLACS3_11125 and LLACS3_11440), mostly at day 1, which may contribute to the initial lipolysis. Indeed, it has been demonstrated that esterases produced by lactic acid bacteria could degrade milk fat into FFAs in hard cheese [69]. Subsequently, few lipase (EC:3.1.1.3) or esterase (EC:3.1.1.13) encoding genes from *G*. *candidum* (i.e., ATG15, TGL1, ROG1) were detected, and their expression level increased along with the ripening time. We also detected several transcripts involved in free fatty acid import including FAA1, FAA2 and FAA4 encoding long-chain fatty acyl-CoA synthetases (EC:6.2.1.3) and PXA1 (synonym: PAT2) and PXA2 (synonym PAT1) encoding peroxisomal fatty acyl-CoA ABC transporters. Furthermore, genes involved in the peroxisomal version of the β-oxidation pathway, namely POX1 (EC:1.3.3.6), CTA1 (EC:1.11.1.6), FOX2 (EC:4.2.1.119), ECI1 (EC:5.3.3.8), SPS19 (EC:1.3.1.34) and POT1 (EC:2.3.1.16), were highly detected from day 7 to day 31.

### Identification of possible biomarkers of the cheese-ripening process by differential expression analysis

*In situ* gene expression measurement is considered as a promising tool for improving our understanding of the microflora activity in cheese and for monitoring the ripening process. Indeed, considerable efforts have been made over the last few years to develop consistent and repeatable methods for *in situ* quantification of mRNA transcripts based on RT-qPCR techniques. Several examples in the context of cheese ripening are now available in the literature [67,70–73]. However, such studies enable the transcription monitoring of a limited number of genes (generally up to one hundred) and, thus, rely on the previous selection of appropriate biomarkers relevant to the targeted functions.

In the following section, we used our metatranscriptomic dataset to select and propose a set of biomarker genes that cover diverse metabolisms relevant in the cheese-ripening process. To do this, we performed differential expression analysis using the DESeq 2 package [31] and first compared the number of differentially expressed genes between each ripening time (Table 3). Based on this result, we decided to separate the ripening kinetics into two phases, the early and late stage of ripening corresponding to day 1 to day 14 (D1 vs. D14) and day 14 to day 31 (D14 vs. D31), respectively, for which differentially expressed genes were observed. The complete lists of gene transcripts showing a differential abundance in these two comparisons, along with their actual adjusted p-values, are available in S4–S5 Tables, respectively. We then manually selected 70 genes from among this list with a high number of reads and related to a biological and/or technological function according to the genome annotations. For the most prominent microorganisms, selected genes are reported in Table 4 and classified into categories that might be relevant for the monitoring of cheese ripening.

**Table 3:** Number of differentially expressed genes according to ripening time.

Day 1	Day 7	Day 14	Day 21	Day 31	
0	302	314	257	203	**Day 1**
	0	56	177	438	**Day 7**
		0	79	482	**Day 14**
			0	330	**Day 21**
				0	**Day 31**

**Table 4:** Selection of genes showing a differential abundance between Day 1 and Day 14 and/or Day 14 and Day 31.

Locus tag (gene)	Product (EC number)	Sepcies	Log2 fold change Day 14 vs. Day 1 (mean)	Log2 fold change Day 31 vs. Day 14 (mean)
***Central Metabolism***				
DEHA2G14058g (ENO1)	Enolase I (EC:4.2.1.11)	*D*. *hansenii*	3.20 (22.81)	-2.36 (21.01)
DEHA2G18348g (PDC1)	Pyruvate decarboxylase (EC:4.1.1.1)	*D*. *hansenii*	2.43 (46.35)	-
DEHA2F04796g (TDH3)	Glyceraldehyde-3-phosphate dehydrogenase (EC:1.2.1.12)	*D*. *hansenii*	2.03 (88.25)	-
DEHA2E13530g (MLS1)	Malate synthase (EC:2.3.3.9)	*D*. *hansenii*	-	1.27 (69.92)
GECA22s00351g (TDH3)	Glyceraldehyde-3-phosphate dehydrogenase (EC:1.2.1.12)	*G*. *candidum*	3.51 (126.71)	-
GECA17s02408g (ENO2)	Enolase II (EC:4.2.1.11)	*G*. *candidum*	3.72 (167.68)	-
GECA06s01693g (MDH1)	Malate dehydrogenase (EC:1.1.1.37)	*G*. *candidum*	4.03 (71.02)	-2.25 (69.13)
GECA06s04762g (PYC2)	Pyruvate carboxylase 2 (EC:6.4.1.1)	*G*. *candidum*	3.24 (200.63)	-1.97 (193.23)
GECA20s01396g (ADH3)	Alcohol dehydrogenase 3 (EC:1.1.1.1)	*G*. *candidum*	-	-1.66 (176.65)
KLLA0A09185g (ENO1)	ENO1 Enolase I (EC:4.2.1.11)	*K*. *lactis*	-6.62 (96.53)	-
KLLA0A11011g (PGK1)	Phosphoglycerate kinase (EC:2.7.2.3)	*K*. *lactis*	-5.58 (119.43)	-
KLLA0A11858g (TDH2)	Glyceraldehyde 3-phosphate dehydrogenase (EC:1.2.1.12)	*K*. *lactis*	-4.68 (184.66)	-
KLLA0E07569g (FBA1)	Fructose 1 6-bisphosphate aldolase (EC:4.1.2.13)	*K*. *lactis*	-2.79 (97.16)	-
KLLA0E16303g (PDC1)	Pyruvate decarboxylase (EC:4.1.1.1)	*K*. *lactis*	-3.38 (163.45)	-
KLLA0F20988g (GAP1)	Glyceraldehyde-3-phosphate dehydrogenase (EC:1.2.1.12)	*K*. *lactis*	-2.47 (80.16)	-3.41 (11.87)
KLLA0F23397g (PYK1)	Pyruvate kinase (EC:2.7.1.40)	*K*. *lactis*	-3.85 (50.88)	-
LLACS3_03095 (gap)	Glyceraldehyde-3-phosphate dehydrogenase (EC:1.2.1.12)	*L*. *lactis*	-5.00 (5185.82)	-
LLACS3_01725 (gap)	Glyceraldehyde-3-phosphate dehydrogenase (EC:1.2.1.12)	*L*. *lactis*	-5.80 (5302.58)	-
LLACS3_09760 (eno)	Enolase (EC:4.2.1.11)	*L*. *lactis*	-6.13 (449.67)	-
LLACS3_02295 (adhE)	Aldehyde-alcohol dehydrogenase (EC:1.1.1.1; EC:1.2.1.10)	*L*. *lactis*	-3.44 (79.61)	-
LLACS3_04185 (gnd)	Phosphogluconate dehydrogenase (EC:1.1.1.44)	*L*. *lactis*	-4.87 (333.29)	-
LLACS3_06550 (deoB)	Phosphopentomutase (EC:5.4.2.7)	*L*. *lactis*	-5.76 (491.42)	-
CCAS_01315 (pyc)	Pyruvate carboxylase (EC:6.4.1.1)	*C*. *casei*	-	4.41 (24.97)
CCAS_05260	2-methylcitrate dehydratase (EC:4.2.1.79)	*C*. *casei*	-	4.96 (118.78)
HALV_03660 (adhE)	Aldehyde-alcohol dehydrogenase (EC:1.1.1.1; EC:1.2.1.10)	*H*. *alvei*	-	3.35 (36.21)
HALV_13325 (fba)	Fructose-bisphosphate aldolase class II (EC:4.1.2.13)	*H*. *alvei*	-	3.56 (73.94)
***Carbohydrate uptake***				
LLACS3_01460 (manX)	PTS system mannose-specific EIIAB component (EC:2.7.1.69)	*L*. *lactis*	-4.54 (72.28)	-
LLACS3_01465 (manY)	PTS system mannose-specific EIIC component	*L*. *lactis*	-4.51 (74.66)	-
LLACS3_11905 (lacE)	PTS system lactose-specific IIBC component (EC:2.7.1.69)	*L*. *lactis*	-3.43 (169.59)	-
LLACS3_11930 (lacA)	Galactose-6-phosphate isomerase subunit lacA (EC:5.3.1.26)	*L*. *lactis*	-3.64 (189.96)	-
LLACS3_07715 (ptsI)	Phosphoenolpyruvate protein phosphotransferase (EC:2.7.3.9)	*L*. *lactis*	-4.95 (2656.44)	-
LLACS3_07720 (ptsH)	Phosphocarrier protein HPr (EC:2.7.11.-)	*L*. *lactis*	-6.80 (2126.77)	-
***Protein degradation***				
GECA06s04927g (PRD1)	Zinc metalloendopeptidase	*G*. *candidum*	6.59 (191.17)	-2.65 (186.53)
GECA01s02760g (PEP4)	Vacuolar aspartyl protease (proteinase A) (EC:3.4.23.25)	*G*. *candidum*	4.26 (118.86)	1.02 (293.28)
GECA10s04102g (CPS1)	Vacuolar carboxypeptidase yscS (EC:3.4.17.4)	*G*. *candidum*	-	-1.58 (62.04)
GECA01s04322g (OPT2)	Oligopeptide transporter	*G*. *candidum*	-	-4.46 (129.07)
LLACS3_02215 (ftsH)	ATP-dependent zinc metalloprotease (EC:3.4.24.-)	*L*. *lactis*	-3.55 (91.99)	-
CCAS_13715	Xaa-Pro dipeptidase (EC:3.4.13.9)	*C*. *casei*	-	2.96 (18.35)
HALV_05100 (hflB)	ATP-dependent metallopeptidase (EC:3.4.24.-)	*H*. *alvei*	-	2.97 (16.07)
HALV_13275 (pepP)	Proline-specific aminopeptidase (EC:3.4.11.5)	*H*. *alvei*	-	4.97 (11.34)
***Amino acid degradation***				
GECA04s04949g (AAT2)	Cytosolic aspartate aminotransferase (EC:2.6.1.1)	*G*. *candidum*	6.10 (76.20)	2.18 (349.16)
GECA22s00494g (CAR2)	L-ornithine transaminase (OTAse) (EC:2.6.1.13)	*G*. *candidum*	-	-2.37 (99.20)
GECA03s01374g (GAP1)	General amino acid permease	*G*. *candidum*	-	-3.74 (113.88)
GECA04s00373g (PPZ2)	Serine/threonine protein phosphatase Z (EC:3.1.3.16)	*G*. *candidum*	-	3.61 (481.29)
GECA05s04498g (DUR1)	Urea amidolyase (EC:6.3.4.6; EC:3.5.1.54)	*G*. *candidum*	-	2.20 (116.57)
GECA20s00296g (LAP3)	Cysteine aminopeptidase (EC:3.4.22.40)	*G*. *candidum*	-	-4.12 (62.59)
GECA06s00802g (GDH2)	NAD(+)-dependent glutamate dehydrogenase (EC:1.4.1.2)	*G*. *candidum*	5.17 (350.98)	-
GECA09s02639g (CYS3)	Cystathionine gamma-lyase (EC:4.4.1.1)	*G*. *candidum*	3.11 (47.57)	-
HALV_00935 (tdcC)	Threonine/serine transporter	*H*. *alvei*	-	4.45 (52.07)
HALV_13395 (speA)	Arginine decarboxylase (EC:4.1.1.19)	*H*. *alvei*	-	3.35 (14.06)
***Sulfur metabolism***				
GECA07s04267g	Putative sulfate permease	*G*. *candidum*	-	1.64 (253.70)
GECA18s00835g (MET6)	Cobalamin-independent methionine synthase (EC:2.1.1.14)	*G*. *candidum*	-	2.44 (255.35)
***Respiration and iron transport***				
DEHA_mCDS7140 (COB)	Apocytochrome b	*D*. *hansenii*	2.96 (99.64)	-
DEHA_mCDS20585 (COX1)	Cytochrome c oxidase subunit 1 (EC:1.9.3.1)	*D*. *hansenii*	2.52 (359.26)	1.11 (818.11)
GECA_mCDS15388 (COB)	Apocytochrome b	*G*. *candidum*	2.39 (598.75)	-0.90 (659.71)
GECA_mCDS24166 (COX1)	Cytochrome c oxidase subunit 1 (EC:1.9.3.1)	*G*. *candidum*	2.77 (841.18)	-
GECA05s03805g (FET3)	Ferro-O2-oxidoreductase	*G*. *candidum*	7.16 (67.98)	-6.71 (58.21)
GECA12s01770g (SIT1)	Ferrioxamine B transporter	*G*. *candidum*	6.14 (112.96)	-4.92 (97.92)
GECA26s00230g (FTR1)	High affinity iron permease	*G*. *candidum*	-	-1.21 (89.06)
KLLA_mCDS20667 (COB)	Apocytochrome b	*K*. *lactis*	-4.74 (431.07)	-
KLLA_mCDS27490 (COX1)	Cytochrome c oxidase subunit 1 (EC:1.9.3.1)	*K*. *lactis*	-4.72 (813.44)	-
CCAS_12555	Iron-siderophore ABC transporter	*C*. *casei*	-	5.99 (53.33)
***Stress response***				
LLACS3_06675 (dnaK)	Chaperone protein DnaK	*L*. *lactis*	-5.94 (10536.90)	1.22 (475.75)
LLACS3_08635 (clpB)	Chaperone protein ClpB	*L*. *lactis*	-5.89 (15396.23)	-
LLACS3_00650 (sodA)	Superoxide dismutase (EC:1.15.1.1)	*L*. *lactis*	-6.40 (6002.79)	-
CCAS_01895	Catalase (EC:1.11.1.6)	*C*. *casei*	-	4.91 (47.50)
GECA07s02177g (RIM101)	Transcriptional repressor involved in response to pH	*G*. *candidum*	-	1.41 (221.55)
***Lipid metabolism***				
GECA13s03354g (GUT1)	Glycerol kinase (EC:2.7.1.30)	*G*. *candidum*	2.49 (147.08)	-2.09 (131.76)
***Endocytosis***				
GECA06s02496g (ALY2)	Alpha arrestin, endocytosis	*G*. *candidum*	-	1.30 (82.97)
***Hyphal growth***				
GECA07s00142g (MIT1)	Transcriptional regulator of pseudohyphal growth	*G*. *candidum*	-	2.17 (82.88)

The log2 fold change and the average number of reads (mean) are indicated only if the gene revealed a significant difference between the two ripening times (adjusted p-value < 0.05).

#### Brief description of differentially expressed genes between D1 and D14

On the basis of the comparative analysis of D1 vs. D14, two genes related to glycolysis, encoding glyceraldehyde-3-phosphate dehydrogenase (EC:1.2.1.12) and enolase (EC:4.2.1.11), could be proposed as common biomarkers of microbial species activity at the early stage of the ripening process. Interestingly, they were more abundant at D1 in *K*. *lactis* and *L*. *lactis*, in agreement with an intense development and metabolic activity for these facultative anaerobic microorganisms during the first days of ripening. As mentioned above, both species are involved in lactose degradation within the cheese matrix. This was exactly the opposite for the cheese-surface aerobic yeasts *G*. *candidum* and *D*. *hansenii* for which both genes were more expressed at D14. Mitochondrial genes of yeasts related to the respiration chain, e.g., COB and COX, followed the same variation pattern.

Regarding *G*. *candidum*, genes involved in protein degradation (e.g., PRD1, PEP4) and in glutamate—the most abundant amino acid—degradation (e.g., GDH2) were more frequently detected at D14 when compared to D1 and could thus be proposed as biomarkers for proteolytic activity and amino acid catabolism. GUT1 encoding glycerol kinase (EC:2.7.1.30) involved in lipid metabolism followed the same trend and could be used as a biomarker for lipolysis.

A striking feature of *L*. *lactis* is the occurrence of several stress-related genes (e.g., *sodA*, *dnaK*, *clpB*) with high expression levels at D1 (S4 Table). This might be related to several possible stresses such as the shift from anaerobic (lactic acid production during the milk acidification and coagulation phase) to aerobic conditions during the ripening process, the osmotic pressure induced by salting or variations in the pH. Indeed, transcriptomic analysis of *L*. *lactis* has already revealed that *sodA* gene overexpression is associated with oxidative stress response during milk fermentation [74], and that genes encoding chaperones induction reflects the growth arrest of *L*. *lactis* in cheese observed almost 24h post-inoculation [75]. In our study, model cheeses were analyzed as a whole, thus including both surface and core parts. However, local conditions might have a great influence on physiochemical stresses encountered by *L*. *lactis* and thus should be taken into account for data interpretation.

#### Brief description of differentially expressed genes between D14 and D31

The comparative analysis of D14 vs. D31 revealed a shift of the metabolism of *G*. *candidum* towards peptide (OPT2, PRD1, LAP3, CPS1) and amino acid (GAP1, AAT2, CAR2) catabolism/transport compared to D1/D14. Another interesting feature is the detection of genes related to iron capture and/or transport, higher in D14 compared to D31 for *G*. *candidum* (SIT1, FET3, FTR1), but lower in *C*. *casei* (iron-siderophore ABC transporter). This indicated that iron capture is crucial for microbial species living on the cheese surface, as demonstrated by Monnet et al. [26]. In *C*. *casei*, we observed the highest abundance at D31 of CCAS_05260 encoding a 2-methylcitrate dehydratase (EC:4.2.1.79), an important enzyme involved in the methylcitrate pathway. In *H*. *alvei*, gene transcripts involved in peptide/amino acid transport (*meoA*, *tdcC*) and degradation (*speA*, *pepP*, *hlfB*) were also more abundant at D31, revealing the catabolic activity of this species at the end of ripening. Finally, two transcripts involved in sulfur metabolism, encoding a putative sulfate permease (GECA07s04267g) and a methionine synthase (MET6), were more highly detected at D31 in *G*. *candidum*. They could be involved in sulfur metabolism through sulfur recycling from various sulfur compounds, including volatile ones, which are released in great quantity at the surface of ripened cheeses [7].

## Conclusion

In this study, we used a combination of microbiological, biochemical, metagenomic and metatranscriptomic methods to obtain a detailed picture of an experimental surface-ripened cheese ecosystem that functions during the ripening process. Overall, we were able to reveal the major contribution of the most dominant microbial species (e.g. *L*. *lactis*, *K*. *lactis*, *G*. *candidum*, *D*. *hansenii* and *C*. *casei*) and possible interactions regarding key functions involved in the dairy matrix degradation. *L*. *lactis* and *K*. *lactis* activities during the early stage of ripening enabled the rapid consumption of lactose. Lactate, produced from lactose by *L*. *lactis*, was then rapidly consumed by *D*. *hansenii* and *G*. *candidum* for which we detected high levels of lactate dehydrogenase transcripts. Regarding protein and lipid metabolism, the great majority of RNA-Seq reads mapped *G*. *candidum* genes, which suggested a strong influence of this species on casein and fat degradation. At the end of ripening, our dataset indicated the expression of amino acid degradation-related genes by *G*. *candidum* and acid-sensitive bacteria such as *C*. *casei* and *H*. *alvei*, which were linked to their late development at the cheese surface. We demonstrated that global gene expression data collected at the ecosystem scale were in good accordance with the observed phenomena (e.g., biochemical and microbiological data) and provided the unique opportunity to simultaneously address questions related to different metabolisms and involving several individual species.

Furthermore, statistical methods based on differential expression analysis made it possible to select a short list of potential biomarkers. This valuable tool might be particularly useful for more precise and in-depth studies aiming at understanding and/or simply following the contribution of different strains or species in the ripening process, sustaining the production of different surface-ripened cheeses.

Altogether, metatranscriptomic analysis revealed the proportion of the genes that are actually expressed within a food microbial community composed of both eukaryotes and prokaryotes. When combined with biochemical data, it may also indicate the microbial populations that are metabolically active and how they respond to a perturbation. Thus, it may be applied to detect near-instantaneous responses to environmental perturbations (e.g. biotic and abiotic constraints) which could occur during the ripening process and other related food fermentation processes. Currently, the main limitation of this approach remains the difficulty to detect low-abundant species which could also contribute to the matrix transformation. The combination of both using efficient rRNA depletion methods and increasing the sequencing depth might enable to partly overcome this limitation.

## Supporting Information

S1 Fig Metabolic pathways detected in the metatranscriptomic dataset. Genes exhibiting an average of > 5 normalized reads were mapped in black onto KEGG general metabolic pathways (ko01100). E: complete ecosystem. LL: *Lactococcus lactis*. KL: *Kluyveromyces lactis*. GC: *Geotrichum candidum*. DH: *Debaryomyces hansenii*. CC: *Corynebacterium casei*. HA: *Hafnia alvei*.
(TIF)

S2 Fig Expression profile of the oxidative phosphorylation pathway during surface-ripened cheese maturation. Histogram charts represent the cumulative number of normalized reads per sampling day and per microbial species. CC: *Corynebacterium casei*, HA: *Hafnia alvei*, AA: *Arthrobacter arilaitensis*, BA: *Brevibacterium aurantiacum*, SE: *Staphylococcus equorum*, LL: *Lactococcus lactis*, KL: *Kluyveromyces lactis*, DH: *Debaryomyces hansenii*, GC: *Geotrichum candidum*.
(TIF)

S3 Fig Expression profile of carbohydrate metabolic pathways. Histogram charts represent the expression dynamics (cumulative number of normalized reads per sampling day and per microbial species) of the galactose metabolism (A), glycolysis-gluconeogenesis pathway (B) and pentose phosphate pathway (C). CC: *Corynebacterium casei*, HA: *Hafnia alvei*, AA: *Arthrobacter arilaitensis*, BA: *Brevibacterium aurantiacum*, SE: *Staphylococcus equorum*, LL: *Lactococcus lactis*, KL: *Kluyveromyces lactis*, DH: *Debaryomyces hansenii*, GC: *Geotrichum candidum*.
(TIF)

S4 Fig Extracellular protease assay. *Yarrowia lipoltica* 1E07 (Yl), *Kluyveromyces lactis* 3550 (Kl), *Debaryomyces hansenii* 304 (Dh) and *Geotrichum candidum* ATCC 204307 (Gc) were spotted on protease assay medium (0.67% yeast nitrogen base without ammonium sulfate and amino acids (Difco Laboratories), 0.1% glucose, 50 mM phosphate buffer, pH 6.8, 2% skim milk (Difco Laboratories)), and incubated 6 days at 18°C. Clarification zone around the colony indicated extracellular protease activity.
(TIF)

S5 Fig Lipolysis index measurement during surface-ripened cheese maturation. (TIF)

S1 Table Metagenome and metatranscriptome statistics. (XLSX)

S2 Table Expression data for all CDSs detected in the metatranscriptome (read numbers were normalized to 50,000 reads per sampling day). (XLSX)

S3 Table Expression data for CDSs encoding peptidases and proteases (read numbers were normalized to 50,000 reads per sampling day). (XLSX)

S4 Table Gene transcripts showing a differential abundance between Day 1 and Day 14. (XLSX)

S5 Table Gene transcripts showing a differential abundance between Day 14 and Day 31. (XLSX)

S1 File Supplementary methods: experimental surface ripened-cheese production. (DOC)
