# Supplementary figures and images for: Overview of a Surface-Ripened Cheese Community Functioning by Meta-Omics Analyses
Source: PLoS One. 2015 Apr 13;10(4):e0124360. doi: 10.1371/journal.pone.0124360 (PMC4395090; doi:10.1371/journal.pone.0124360)

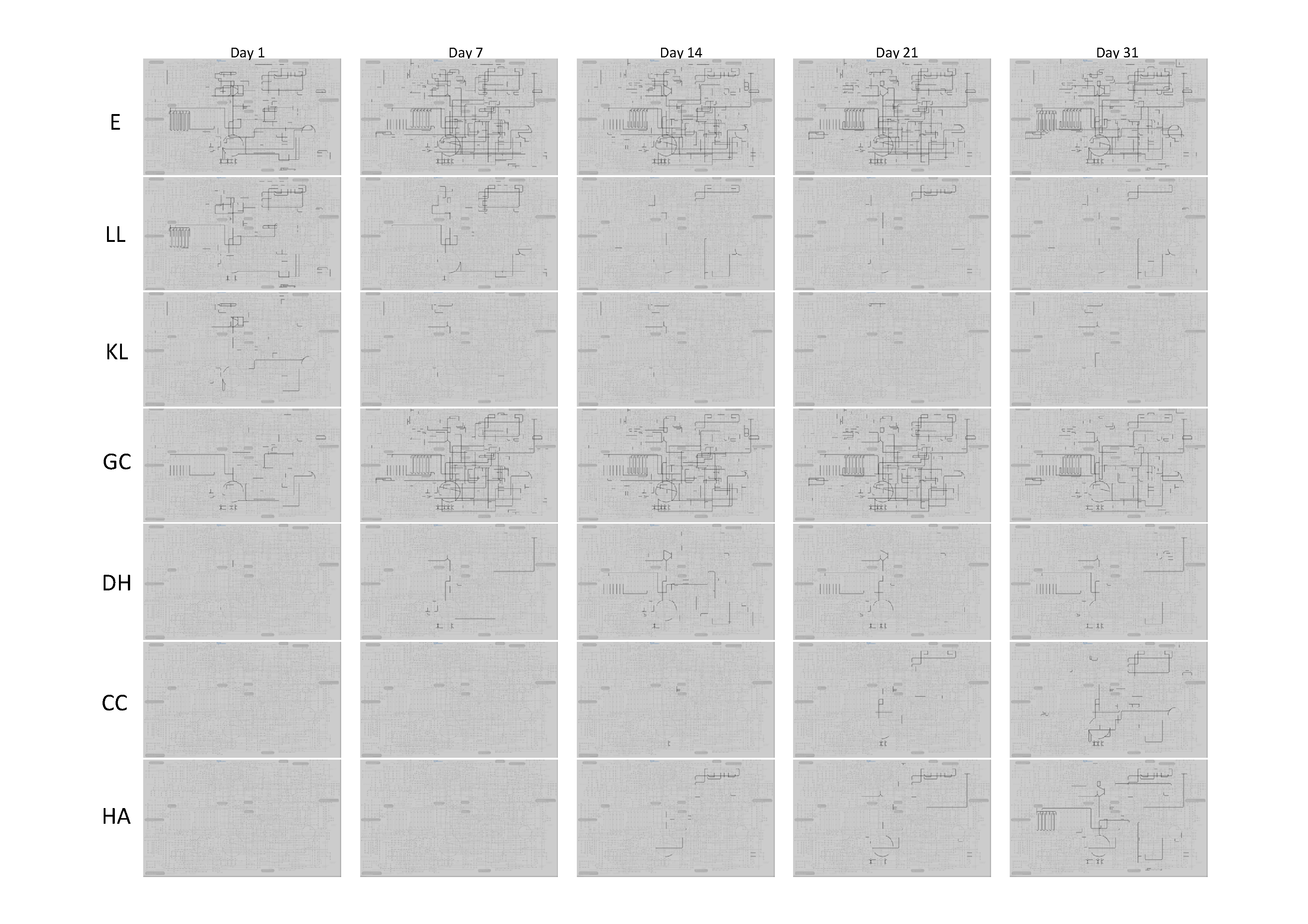

Supplement: S1 Fig — Genes exhibiting an average of > 5 normalized reads were mapped in black onto KEGG general metabolic pathways (ko01100). E: complete ecosystem. LL: Lactococcus lactis. KL: Kluyveromyces lactis. GC: Geotrichum candidum. DH: Debaryomyces hansenii. CC: Corynebacterium casei. HA: Hafnia alvei. (TIF) [file pone.0124360.s001.tif]

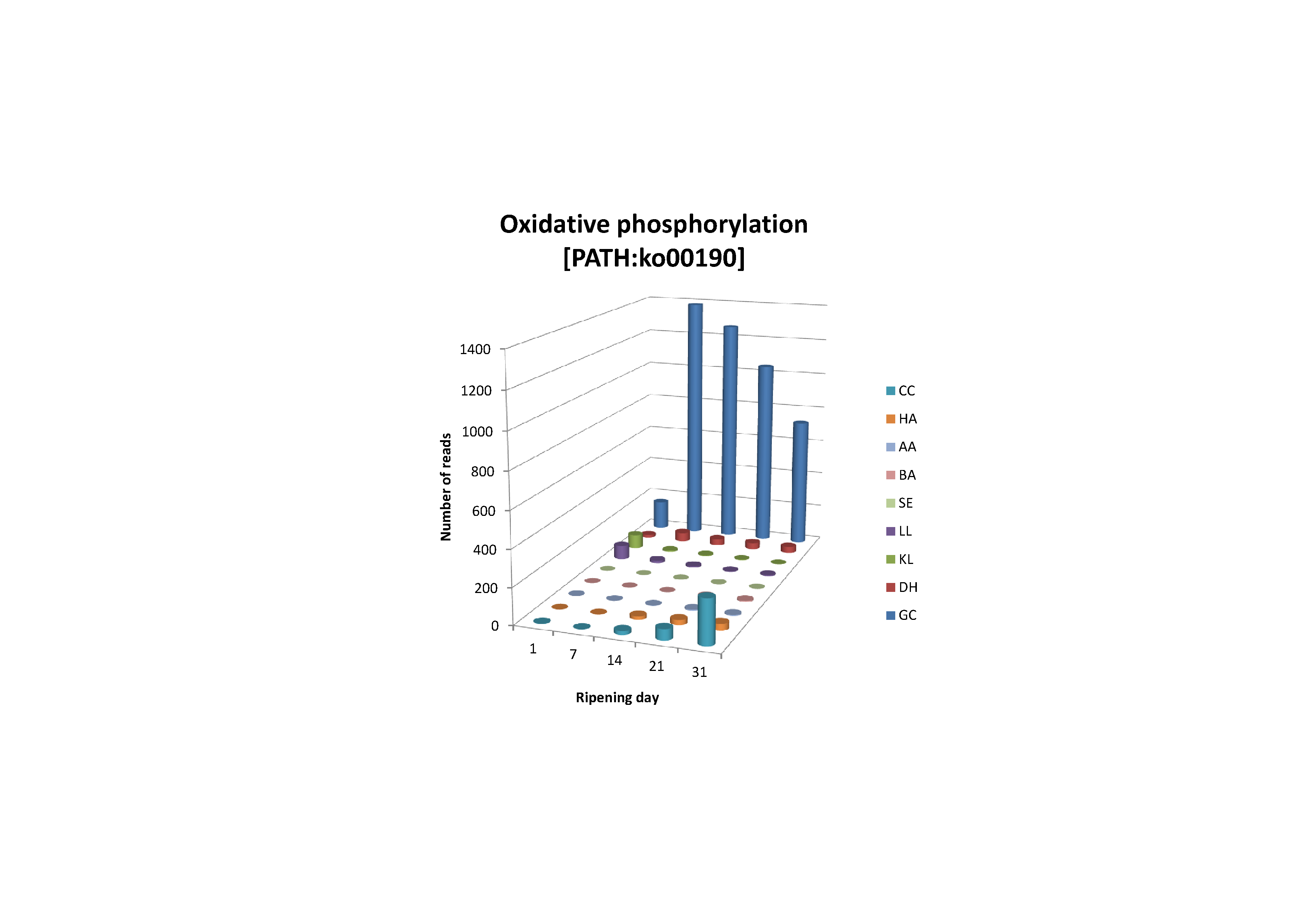

Supplement: S2 Fig — Histogram charts represent the cumulative number of normalized reads per sampling day and per microbial species. CC: Corynebacterium casei, HA: Hafnia alvei, AA: Arthrobacter arilaitensis, BA: Brevibacterium aurantiacum, SE: Staphylococcus equorum, LL: Lactococcus lactis, KL: Kluyveromyces lactis, DH: Debaryomyces hansenii, GC: Geotrichum candidum. (TIF) [file pone.0124360.s002.tif]

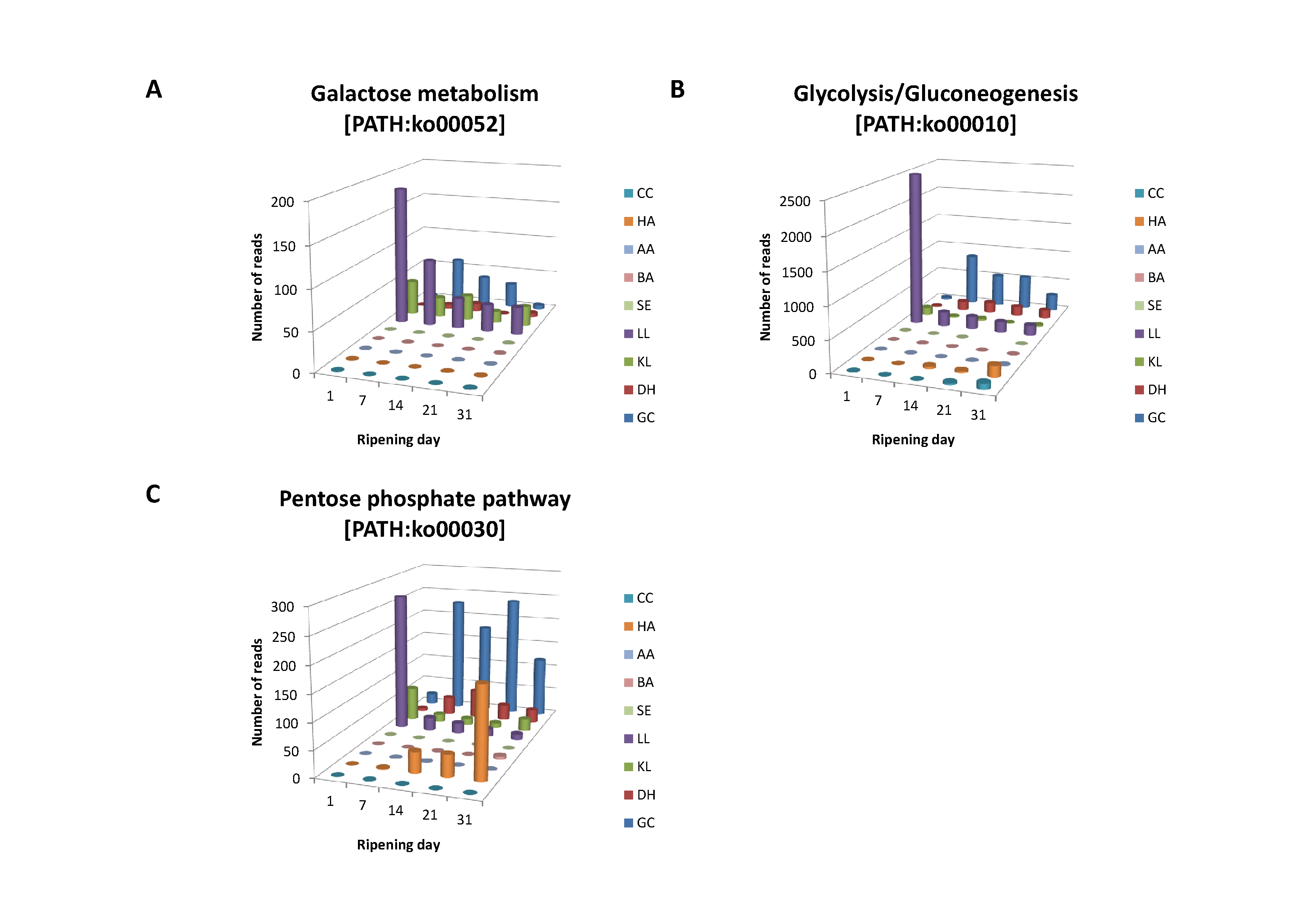

Supplement: S3 Fig — Histogram charts represent the expression dynamics (cumulative number of normalized reads per sampling day and per microbial species) of the galactose metabolism (A), glycolysis-gluconeogenesis pathway (B) and pentose phosphate pathway (C). CC: Corynebacterium casei, HA: Hafnia alvei, AA: Arthrobacter arilaitensis, BA: Brevibacterium aurantiacum, SE: Staphylococcus equorum, LL: Lactococcus lactis, KL: Kluyveromyces lactis, DH: Debaryomyces hansenii, GC: Geotrichum candidum. (TIF) [file pone.0124360.s003.tif]

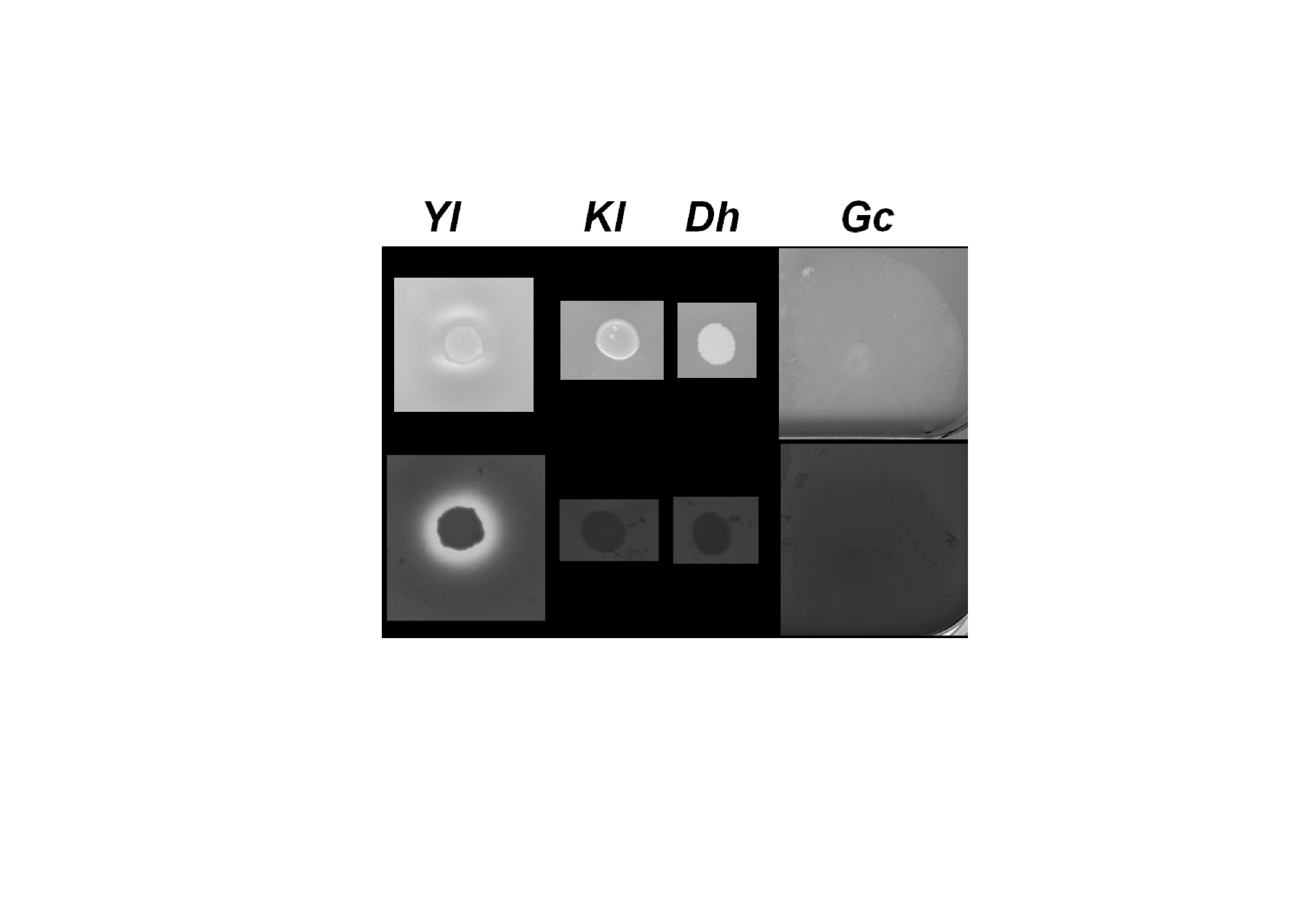

Supplement: S4 Fig — Yarrowia lipoltica 1E07 (Yl), Kluyveromyces lactis 3550 (Kl), Debaryomyces hansenii 304 (Dh) and Geotrichum candidum ATCC 204307 (Gc) were spotted on protease assay medium (0.67% yeast nitrogen base without ammonium sulfate and amino acids (Difco Laboratories), 0.1% glucose, 50 mM phosphate buffer, pH 6.8, 2% skim milk (Difco Laboratories)), and incubated 6 days at 18°C. Clarification zone around the colony indicated extracellular protease activity. (TIF) [file pone.0124360.s004.tif]

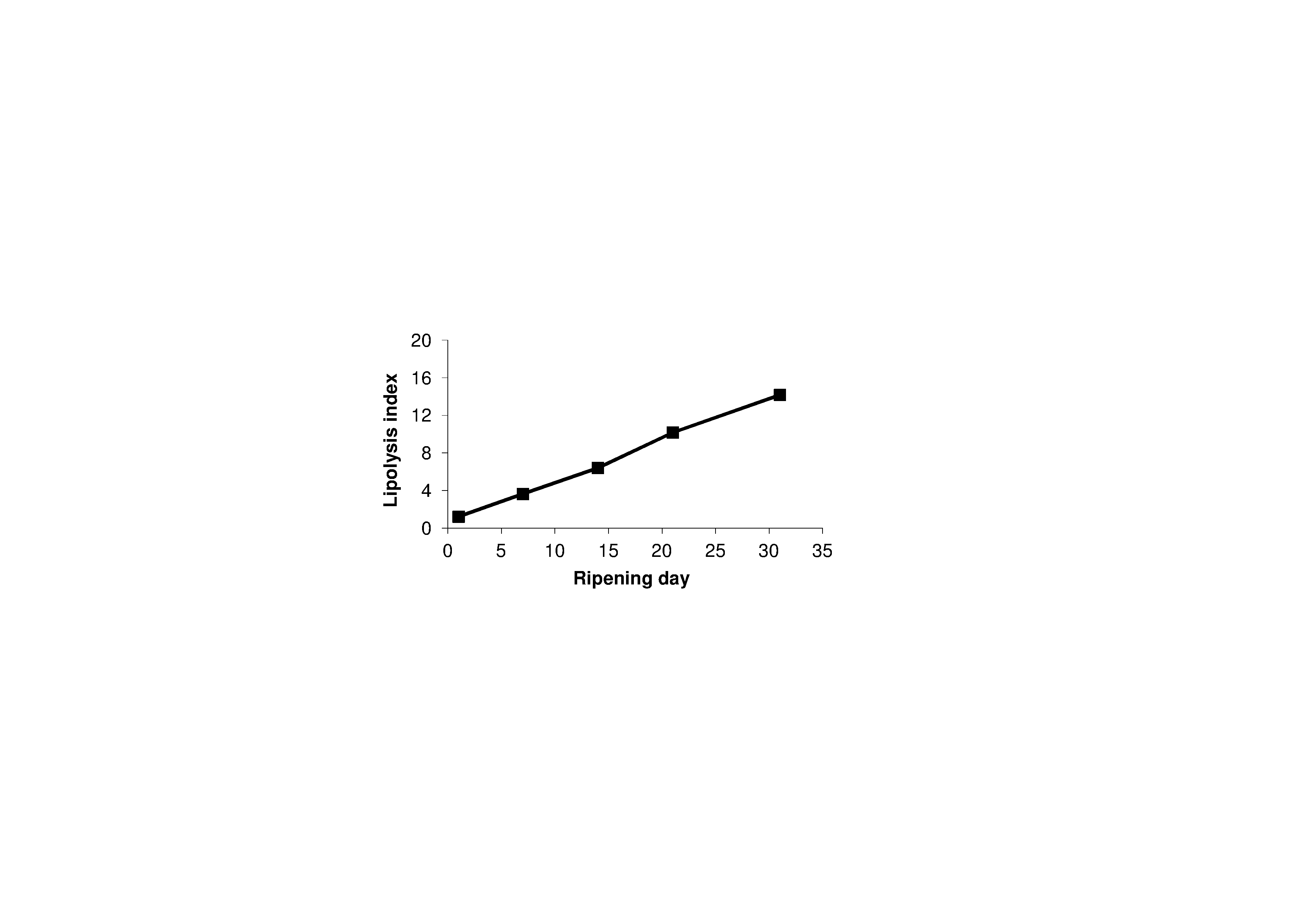

Supplement: S5 Fig — (TIF) [file pone.0124360.s005.tif]
