# Supplementary material for: Overview of a Surface-Ripened Cheese Community Functioning by Meta-Omics Analyses
Source: PLoS One. 2015 Apr 13;10(4):e0124360. doi: 10.1371/journal.pone.0124360 (PMC4395090; doi:10.1371/journal.pone.0124360)
Supplement: S1 File — (DOC) [file pone.0124360.s011.doc]

**Supplementary methods: experimental surface ripened-cheese production.**

***Strains and growth conditions****.*The nine microorganisms that composed the model microbial community were *Corynebacterium casei* UCMA 3821 (obtained from the Laboratoire des Micro-organismes d'Intérêt Laitier et Alimentaire (Caen, France)), *Brevibacterium aurantiacum* ATCC 9174, *Geotrichum candidum* ATCC 204307 (both from the American Type Culture Collection (Rockville, MD, USA)), *Arthrobacter arilaitensis* CIP 108037 (from the Collection de l'Institut Pasteur (Paris, France)), *Staphylococcus equorum* Mu2, *Hafnia alvei* GB001, *Lactococcus lactis* subsp. *lactis* S3+ and its protease-negative variant S3-, *Kluyveromyces lactis* 1 and *Debaryomyces hansenii* 304 (from the Génie et Microbiologie des Procédés Alimentaires culture collection (INRA, Thiverval-Grignon, France)). All these strains were originally isolated from cheeses. The lactic acid bacteria S3+ and S3- were grown for 24 h at 30°C under static conditions in M17 lactose (0.5%) broth (Biokar Diagnostics, Beauvais, France), inoculated at 3% in reconstituted skimmed milk (100 g/L, Difco Laboratories, Detroit, MI, USA) and then incubated for 16 h at 30°C. All other bacteria were grown under aerobic conditions (using a rotary shaker at 150 rpm) at 25°C for 48 h in 50 mL conical flasks containing 10 mL of brain heart infusion broth (Biokar Diagnostics). The yeasts were grown under the same conditions, except that potato dextrose broth (Biokar Diagnostics) was used as the growth medium.

***Cheese production.*** Pilot-scale Livarot-type cheese production (coagulation, cutting, draining, and moulding of the curd) was carried out under aseptic conditions in a sterilized 3 m^3^ chamber [20]. The milk used (120 L) was obtained from an experimental farm (AgroParisTech, Thiverval-Grignon, France) and standardized at a fat content of 29 g/L by mixing skimmed and full-cream milk. The milk was pasteurized for 2.5 min at 77°C. After a few L of milk had been pumped into the tank, the batch was inoculated with *Lactococcus lactis* subsp. *lactis* S3+ and S3- at concentrations equivalent (for 120 L of milk) to 2 x 10^6^ and 4 x 10^6^ CFU/mL, respectively. The yeast cells grown in potato dextrose broth were recovered by centrifugation and then washed and resuspended in physiological saline solution (NaCl, 9 g/L). *Kluyveromyces lactis* and *Debaryomyces hansenii* were inoculated at 10^4^ CFU/mL, and *Geotrichum candidum* was inoculated at 10^3^ CFU/mL. Next, 120 mL of a filter-sterilized CaCl_2_ solution (10%) were added. Once the pH reached a value of 6.3, 40 mL of the coagulant (containing 520 mg/L of chymosin (Chr. Hansen, Arpajon, France)) were added. The rennet was allowed to coagulate for 20 min and the curd was cut after 40 min of hardening. After the curd had set for 5 min, 60 L of whey were removed prior to moulding. The cheeses were shaped in circular moulds (diameter: 20 cm; height: 15 cm). The moulds were incubated at 26°C, inverted three times (after 30 min, 2 h and 5 h) and demoulded after 21 h.

***Cheese cutting and salting.*** Under aseptic conditions, the cheeses were cut into smaller cheeses (diameter: 5 cm; height: 1.5 cm) using a knife and a circular punch. Each of these small cheeses weighed approximately 26 g. They were then immersed for 3 min in sterile brine (270 g/L NaCl) at 14°C. This resulted in a final salt concentration of ~1.5%. The cheeses were then transferred into sterile crystallizing basins (diameter: 13.5 cm) and incubated for 24 h at 21°C and 93% relative humidity.

***Smearing.*** Liquid cultures of the five ripening bacterial strains were centrifuged for 10 min at 4,500 g and 4°C. The supernatants were discarded and the cells were resuspended in physiological saline solution to obtain a concentration of 10^10^ CFU/mL. Next, 380 µL of each of the five suspensions were added to 190 mL of physiological saline solution, yielding a smearing solution in which the concentration of each species of bacterium was 2 x 10^7^ CFU/mL. With a sterilized paintbrush, 300 µL of this solution were spread onto the whole surface of each cheese (corresponding to an inoculation level of ~ 2.3 x 10^5^ CFU/g cheese). The inoculated cheeses were transferred into sterile crystallizing basins (four cheeses per basin) and ripened for four weeks at 14°C and 97% relative humidity.
